# Evaluation of silica nanoparticle binding to major human blood proteins

**DOI:** 10.1186/1556-276X-9-668

**Published:** 2014-12-11

**Authors:** Katsutomo Hata, Kazuma Higashisaka, Kazuya Nagano, Yohei Mukai, Haruhiko Kamada, Shin-ichi Tsunoda, Yasuo Yoshioka, Yasuo Tsutsumi

**Affiliations:** Laboratory of Toxicology and Safety Science, Graduate School of Pharmaceutical Sciences, Osaka University, 1-6 Yamadaoka, Suita, Osaka, 565-0871 Japan; Laboratory of Biopharmaceutical Research, National Institute of Biomedical Innovation, 7-6-8, Saito-Asagi, Ibaraki, Osaka, 567-0085 Japan; Laboratory of Innovative Antibody Engineering and Design, Center for Drug Innovation and Screening, National Institute of Biomedical Innovation, 7-6-8 Saito-Asagi, Ibaraki, Osaka, 567-0085 Japan; The Center for Advanced Medical Engineering and Informatics, Osaka University, 1-6, Yamadaoka, Suita, Osaka, 565-0871 Japan

**Keywords:** Nanomaterials, Protein corona, Biological interaction

## Abstract

Nanomaterials are used for various biomedical applications because they are often more effective than conventional materials. Recently, however, it has become clear that the protein corona that forms on the surface of nanomaterials when they make contact with biological fluids, such as blood, influences the pharmacokinetics and biological responses induced by the nanomaterials. Therefore, when evaluating nanomaterial safety and efficacy, it is important to analyze the interaction between nanomaterials and proteins in biological fluids and to evaluate the effects of the protein corona. Here, we evaluated the interaction of silica nanoparticles, a commonly used nanomaterial, with the human blood proteins albumin, transferrin, fibrinogen, and IgG. Sodium dodecyl sulfate-polyacrylamide gel electrophoresis analysis showed that the amount of albumin, transferrin, and IgG binding to the silica particles increased as the particle size decreased under conditions where the silica particle mass remained the same. However, under conditions in which the specific surface area remained constant, there were no differences in the binding of human plasma proteins to the silica particles tested, suggesting that the binding of silica particles with human plasma proteins is dependent on the specific surface area of the silica particles. Furthermore, the amount of albumin, transferrin, and IgG binding to silica nanoparticles with a diameter of 70 nm (nSP70) and a functional amino group was lower than that with unmodified nSP70, although there was no difference in the binding between nSP70 with the surface modification of a carboxyl functional group and nSP70. These results suggest that the characteristics of nanomaterials are important for binding with human blood proteins; this information may contribute to the development of safe and effective nanomaterials.

## Background

The development and use of materials at the nanometer scale already have multiple applications in various fields, including the food and cosmetics industries; such nanomaterials have thus become indispensable in our lives [1, 2]. Silica nanoparticles have been widely used in many consumer products because of their useful properties, including relatively low production costs, easy separation, and ease of modification of their surface properties. Accordingly, they have attracted the attention of the pharmaceutical industry as materials for new drug delivery and diagnostic systems [3]. However, because nanomaterials possess novel properties that are different from conventional materials, concerns about their potential unanticipated effects have been raised. We previously found that silica nanoparticles with a diameter of ≤100 nm could cause pregnancy complications [4] or consumptive coagulopathy in mice after systemic exposure [5] compared with silica particles with a diameter of >100 nm. We also demonstrated that the surface-modified silica nanoparticles were unlikely to induce undesired inflammatory responses *in vitro* and *in vivo*, suggesting that it might be possible to decrease the adverse biological effects of nanomaterials and enhance nanomaterial safety by modifying their surface properties [6, 7].

Nanomaterial-mediated biological effects are related to physical characteristics such as particle size and the surface properties of the nanomaterials. When nanomaterials interact with proteins, a protein layer forms around the nanomaterials, referred to as the protein corona, which is dependent on the physical characteristics of the nanomaterial [8, 9]. Many studies have shown that this protein corona plays a role in the biological effects induced by the nanomaterial and in the *in vivo* and *in vitro* kinetics of the nanomaterial. For example, Jiang et al. demonstrated that the uptake of FePt nanoparticles by HeLa cells is suppressed by the adsorption of human transferrin compared with that of the bare nanoparticles [10]. Similarly, the work of Ge et al. suggests that the binding of blood proteins to carbon nanotubes reduces those nanotubes’ cytotoxicity [11]. Therefore, it is important to understand how nanomaterials interact with proteins to clarify both the biodistribution and safety of such nanomaterials. Although proteomics studies have comprehensively identified the blood proteins that make direct contact with nanomaterials, detailed analyses of the binding to individual blood proteins are incomplete lacking. To fully realize the potential of the protein corona, it is essential to understand and quantify the effects of individual proteins on the characteristics of nanomaterials. Furthermore, analyses of the amount of binding of each individual human plasma protein to nanomaterials with various properties would be invaluable in developing the safety profile of such nanomaterials.

In this study, we assess the differences in the interactions between major blood proteins and silica nanoparticles by changing nanomaterial characteristics such as size, specific surface area, and surface modification.

## Methods

### Materials

Human albumin (molecular weight 66 kDa), human fibrinogen (molecular weight 340 kDa), and human transferrin (molecular weight 78 kDa) were purchased from Wako (Osaka, Japan). Human immunoglobulin G (IgG) (molecular weight 146 kDa) was purchased from Oriental Yeast Co. Ltd. (Tokyo, Japan) and human α1-acid glycoprotein (AGP) (molecular weight 44.1 kDa) was purchased from Sigma-Aldrich (Saint Louis, MO, USA).

### Silica particles

Silica particles were purchased from Micromod Partikeltechnologie (Rostock/Warnemünde, Germany). Silica particles with diameters of 70, 100, 300, and 1,000 nm (nSP70, nSP100, nSP300, and mSP1000, respectively) and nSP70 with the surface modification of an added amino group (nSP70-N) or an added carboxyl group (nSP70-C) were used in this study. These particles were sonicated for 5 min and vortexed for 1 min prior to use.

### Recovery of silica particles

Suspensions of silica particles (200 μL) were centrifuged at 21,500 × *g* for 20 min at 4°C. The pellets were washed three times with 500 μL of phosphate buffered saline (PBS) and then dissolved in 200 μL of 5% HNO_3_ (Nacalai Tesque, Kyoto, Japan). The solutions (100 μL) were then transferred to centrifugation tubes and 5 mL of Milli-Q water (Millipore, Billerica, MA, USA) was added to each tube. The silicon content of the samples was analyzed by using inductively coupled plasma mass spectrometry (ICP-MS) (Agilent Technologies, Santa Clara, CA, USA). The recovery of the silica particles was calculated as the ratio of the silicon signal (cps) of the centrifuged samples to that of the uncentrifuged samples.

### Gel electrophoresis

Each protein solution (200 μL), adjusted with PBS to obtain concentrations commonly found in human blood (albumin, 40 mg/mL; fibrinogen, 2 mg/mL; transferrin, 2 mg/mL; IgG, 10 mg/mL; and AGP, 0.5 mg/mL), was mixed with nSP70, nSP100, nSP300, mSP1000, nSP70-C, or nSP70-N (25 mg/mL) at 1:1 (*v*/*v*). To assess the effect of the specific surface area, each protein solution was mixed with nSP70 (1.08 × 10^6^ mm^2^/mL), nSP100 (7.54 × 10^5^ particles/mL), nSP300 (2.54 × 10^5^ mm^2^/mL), or mSP1000 (7.54 × 10^4^ particles/mL) at 1.75, 2.5, 7.41, and 25 mg/mL, respectively, which is about 1.5 × 10^4^ mm^2^ of specific surface area for each particle. The mixtures were then vortexed and incubated at 37°C for 1 h with constant rotating. The proteins bound to silica particles were separated by centrifugation at 21,500 × *g* for 20 min at 4°C. The pellets were washed three times with 500 μL of PBS to remove the unbound proteins. Proteins bound to the silica particles were suspended in 10% sodium dodecyl sulfate (SDS) buffer (sample solution). Then, the sample solution and Laemmli sample buffer (Bio-Rad Laboratories, Hercules, CA, USA) were mixed in equal amounts (mixture solution) and boiled for 5 min at 95°C. The IgG samples were diluted tenfold before being mixed with the sample buffer because their concentrations were too high to analyze the bands clearly. Each sample was separated by SDS polyacrylamide gel electrophoresis (PAGE) (ATTO, Tokyo, Japan). For the analysis of albumin, transferrin, and IgG, 10% to 20% e-PAGEL was used; for fibrinogen, 5% e-PAGEL was used. Electrophoresis was performed at 15 mA/gel for 10 min (stacking), followed by separation (600 V, 40 mA/gel) for approximately 45 min. After the electrophoresis, the gel was stained by Coomassie brilliant blue (CBB) staining, and the protein bands were quantified with the ImageJ software (http://rsb.info.nih.gov/ij/). The protein content of each band was estimated from its optical density compared with that of each standard solution. As standard solutions, the following protein concentrations were prepared: albumin at 0.08, 0.16, 0.4, and 0.8 mg/mL; transferrin at 0.2, 0.4, 1.0, and 2.0 mg/mL; AGP at 0.05, 0.1, 0.25, and 0.5 mg/mL; IgG at 0.1, 0.2, 0.5, and 1.0 mg/mL; and fibrinogen at 0.2, 0.4, 1.0, and 2.0 mg/mL. The degree of binding was calculated by taking the theoretical concentrations calculated from the proteins added to the silica particles originally as 100%. Values less than the detection limit of the standard curve for each protein were recorded as 0%, even if bands were slightly detectable.

### Statistical analyses

All results are expressed as means ± SD. Differences were compared by means of Tukey’s multiple comparison test.

## Results

### Recovery of silica particles after centrifugation

In this study, we used silica particles with diameters of 70 nm (nSP70), 100 nm (nSP100), 300 nm (nSP300), and 1,000 nm (mSP1000), and nSP70 surface-modified with a carboxy group (nSP70-C) or an amino group (nSP70-N). The physical characteristics of these particles were assessed in previous studies [4, 12]. Transmission electron microscopy analysis showed that they were smooth-surfaced spheres. Dynamic light scattering analysis revealed that the hydrodynamic diameters were 76, 106, 264, and 1136 nm for nSP70, nSP100, nSP300, and mSP1000 in PBS (pH7.4), respectively, and that the zeta potential of each particle was -19.5, -24.3, -25.8, and -33.2 mV, respectively. In addition, we previously reported that the mean secondary particle size of nSP70-C and nSP70-N was 69.6 and 71.8 nm, respectively, and that the zeta potentials were -76.3 and -29.0 mV, respectively. The size distribution spectrum of each set of silica particles showed a single peak, and the measured hydrodynamic diameter corresponded almost precisely to the primary particle size of each set of silica particles. These results indicate that the silica particles used in this study were well-dispersed in solution.

First, we wanted to evaluate the binding of silica particles with the major blood proteins after they were mixed and then centrifuged. Therefore, it was essential to assess how much of each silica particle was precipitated after centrifugation. We analyzed the recovery of each silica particle after centrifugation by using ICP-MS. ICP-MS analysis showed that the percentage recovery of nSP70, nSP70-N, nSP70-C, nSP100, nSP300, and mSP1000 was 71.0, 78.5, 71.5, 80.9, 82.6, and 77.2, respectively. These results show that the amounts of each silica particle recovered were similar, indicating that it would be appropriate to directly compare the degree of binding of each silica particle to the proteins.

### Effects of particle size on binding to proteins

**Figure 1:**
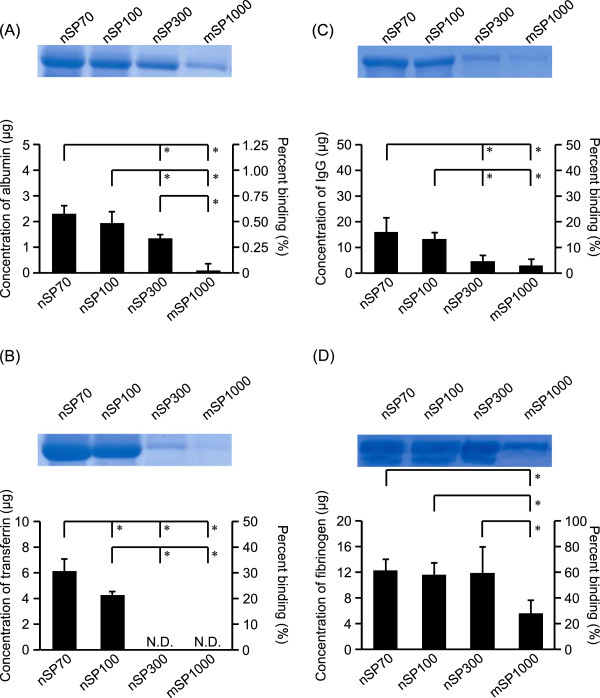
**Effects of silica particle size on binding to human plasma proteins.** Each protein solution was mixed with SP70, nSP100, nSP300, or mSP1000 (25 mg/mL). After centrifugation, each sample was separated by SDS-PAGE. The gel was stained by CBB staining, and the protein bands of albumin **(A)**, transferrin **(B)**, IgG **(C)**, and fibrinogen **(D)** were quantified with the ImageJ software. The protein content of each band was estimated from its optical density compared with the optical density of each standard solution. Data are presented as the mean ± SD; *n* = 4; **P < 0.01 vs. nSP70-treated group; *P < 0.05 vs. nSP70-treated group; N.D., not detected.

Next, we evaluated whether the differences in the size of the silica particles affected their binding to albumin, transferrin, fibrinogen, and IgG. We selected these proteins because these have high concentrations in human blood [13] and because the frequency of contact in the body is considered to be high. In addition, it was reported that these proteins were identified in protein corona on silica nanoparticles with human plasma [14, 15]. SDS-PAGE analysis showed that there was no interaction between the silica particles and AGP (data not shown). For albumin (Figure 1A) and IgG (Figure 1C), the binding was dependent on the diameter of the silica particles. For transferrin, we found that nSP300 and mSP1000 did not bind (Figure 1B). In addition, the degree of binding of mSP1000 with fibrinogen was significantly lower than that of the other particles (Figure 1D). These results indicate that the binding of silica particles to albumin and IgG appears to increase as the particle size decreases. The degree of nSP70 binding was greatest with fibrinogen and smallest with albumin among these four proteins tested.

**Figure 2:**
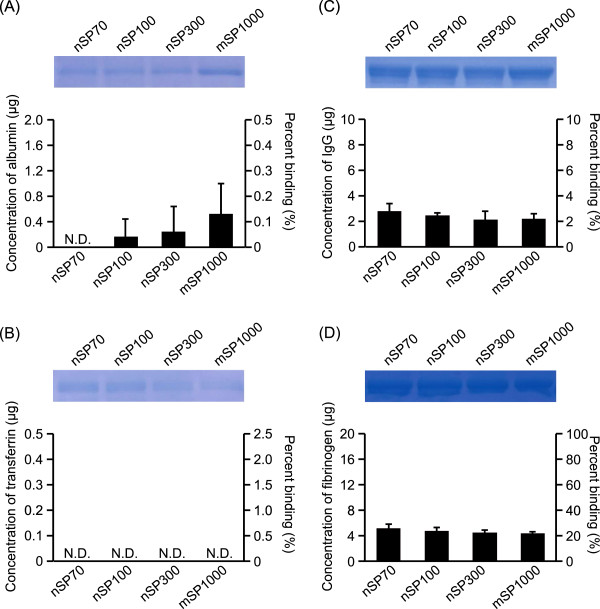
**Effects of the specific surface area of silica particles on binding to human plasma proteins.** Each protein solution was mixed with SP70, nSP100, nSP300, or mSP1000 at 1.75, 2.5, 7.41, and 25 mg/mL, respectively (i.e., approximately 1.5 × 10^4^ mm^2^ of specific surface area). After centrifugation, each sample was separated by SDS-PAGE. The gel was stained by CBB staining, and the protein bands of albumin **(A)**, transferrin **(B)**, IgG **(C)**, and fibrinogen **(D)** were quantified with the ImageJ software. The protein content of each band was estimated from its optical density compared with the optical density of each standard solution. Data are presented as the mean ± SD; *n* = 3; **P < 0.01 vs. nSP70-treated group; *P < 0.05 vs. nSP70-treated group; N.D., not detected.

We also evaluated the effect of the specific surface area of the silica particles on binding to human plasma proteins. The specific surface areas of each particle were matched with that of 25 mg/mL of mSP1000 (about 1.5 × 10^4^ mm^2^), because mSP1000 was the largest particle in this study. The results showed that there was no significant difference in binding with each protein among each size of silica particle tested (Figure 2A,B,C,D), suggesting that the binding of silica particles to human plasma proteins is dependent on the specific surface area of the silica particles.

### Effects of particle surface charge on binding to proteins

**Figure 3:**
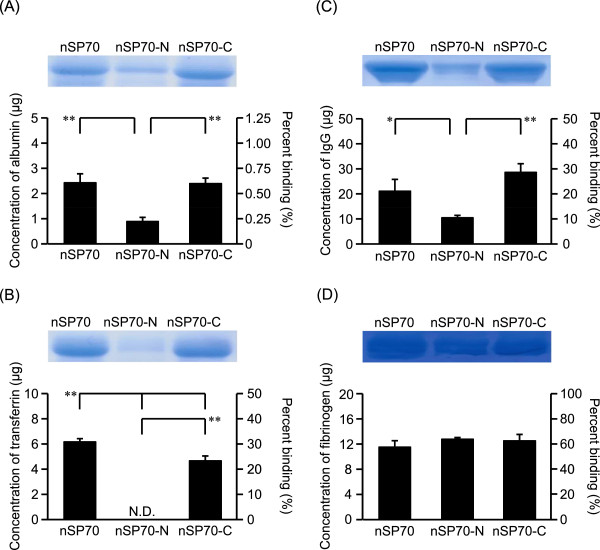
**Effects of surface modification of silica particles on binding to human plasma proteins.** Each protein solution was mixed with SP70, nSP70-N, or nSP70-C (25 mg/mL). After centrifugation, each sample was separated by SDS-PAGE. The gel was stained by CBB staining, and the protein bands of albumin **(A)**, transferrin **(B)**, IgG **(C)**, and fibrinogen **(D)** were quantified with the ImageJ software. The protein content of each band was estimated from its optical density compared with the optical density of each standard solution. Data are presented as the mean ± SD; *n* = 4; **P < 0.01 vs. nSP70-treated group; *P < 0.05 vs. nSP70-treated group; N.D., not detected.

The physical properties of nanomaterials, including surface properties and morphology, are important factors in the biological responses to these materials. We previously demonstrated that nSP70 surface-modified with a carboxyl or amino group might induce abnormal activation of the coagulation cascade to a lesser extent than does unmodified nSP70 [12] and that differences in the properties of surface-modified silica nanoparticles directly affect the extent to which these particles interact with blood proteins, such as coagulation factor XII (unpublished data). Here, we assessed how surface modification affected the protein binding with silica nanoparticles. The surface modification of adding an amino group weakened the binding of nSP70 to albumin (Figure 3A), transferrin (Figure 3B), and IgG (Figure 3C), although the surface modification of adding the carboxyl-group made no noticeable difference to the binding of nSP70 to albumin and IgG. Similarly, there was no difference in fibrinogen binding among these three silica nanoparticles, suggesting that the physical properties associated with nanoparticle interaction with proteins are dependent on the kind of protein involved.

## Discussion

Here, we assessed the effect of nanoparticle size on binding to proteins. We found that the binding of silica particles to albumin, transferrin, and IgG appears to increase as the particle size decreased (Figure 1A,B,C). However, we found no significant difference in binding to these proteins when the specific surface area of the different silica particles was the same (Figure 2A,B,C). The larger specific surface area of nSP70 relative to that of nSP300 and mSP1000 may have allowed the smaller particles to interact with biomolecules such as proteins. These results indicate that specific surface area plays a role in determining silica nanoparticle-protein interactions. How specific surface area influences nanoparticle binding to plasma proteins is poorly understood [16]. Several studies have shown that biological responses to nanomaterials could depend on specific surface area rather than on the mass of the particle [17]. Therefore, biological responses should be assessed in terms of the nanomaterial specific surface area-protein interaction.

The surface charge of the nanomaterials affects the composition of the protein corona. We demonstrated that the surface modification of adding an amino group to nSP70 weakens the binding of nSP70 to albumin, transferrin, and IgG, although there were no significant differences between the binding of nSP70 and that of nSP70 with the surface modification of an added carboxyl group (Figure 3A,B,C). One study showed that polystyrene nanoparticles with a positively charged functional group were more likely to adsorb to proteins with an isoelectric point (pI) ≤5.5, whereas polystyrene nanoparticles with a negatively charged functional group were more likely to adsorb to proteins with a pI >5.5, and the distinct proteins appeared to show a preference for different functional groups [18]. Our results showed that the binding of nSP70-N with albumin (pI 5.92), transferrin (pI 6.81), and IgG (pI 6.4 to 6.9) was reduced relative to that of nSP70. It is conceivable that nSP70-N displayed a positive charge as - NH_3_^+^ in solution and that it did not show any preference for proteins with a pI >5.5. However, there was no significant difference in binding to fibrinogen (pI 5.7) between nSP70 and nSP70-N (Figure 3D). Fibrinogen binds to a wide variety of nanomaterials because it has several different binding domains that accommodate different nanomaterials [19]. Therefore, to understand the differences in the interactions between blood proteins and nanomaterials, it is essential to assess not only the characteristics of the nanomaterials but also the physical properties of the absorbed proteins such as their molecular weight, pI, and structure.

According to the technical data sheets posted in Micromod Partikeltechnologie, the nSP70 in this study became covered with silanol groups and was modified by the addition of an amino or a carboxyl group through its spacer structure. It is difficult to simply compare the difference between nSP70 and modified-nSP70 in terms of their interactions with proteins, but we believe that a difference in the binding domain affinities of the respective particles resulted in the difference in binding between nSP70 and nSP70-N.

The binding of protein with nanomaterials might play important roles in the ultimate fate of these nanomaterials in the body and in the subsequent nanomaterial-induced biological effects [20]. In this study, we showed that the degree of nSP70 binding to fibrinogen was greater than that to the other proteins tested (Figure 1A,B,C,D). The adsorption of opsonins such as fibrinogen and IgG creates a molecular signature that is recognized by immune cells and determines the route of particle internalization. This promotes phagocytosis and removal of the particles from the systemic circulation by cells of the mononuclear phagocytic systems [16, 21]. Moreover, the nanoparticle-induced unfolding of fibrinogen has been shown to promote inflammation via Mac-1 receptor activation [17]. These data and our current results showing that the binding of nSP70 to fibrinogen was greater than that to other proteins are consistent with our previous finding that silica nanoparticles induce strong inflammatory responses [7]. We believe that this study on the interactions of nanomaterials with individual proteins provides essential information for evaluating the biological responses to nanomaterials and that it could lead to the development of safer and more efficacious nanomaterials.

## Conclusions

Here, we showed that the binding of silica nanoparticles with albumin, transferrin, IgG, and fibrinogen is dependent on the specific surface area, not the size of the silica nanoparticles. We also showed that the binding rates of nSP70-N to albumin, transferrin, and IgG were weaker than that of nSP70 and nSP70-C; however, there was no difference in fibrinogen binding among nSP70, nSP70-N, and nSP70-C. We expect that it was connected for the development and use of safer and more effective nanomaterials by understanding the interaction between the nanomaterials and the proteins.

## Abbreviations

AGP: α1-acid glycoprotein
CBB: Coomassie brilliant blue
ICP-MS: inductively coupled plasma mass spectrometry
IgG: immunoglobulin
nSP70: 70-nm-diameter silica nanoparticles
nSP70-C: nSP70 surface-modified with carboxyl groups
nSP70-N: nSP70 surface-modified with amino groups
nSP300: 300-nm-diameter silica particles
mSP1000: 1000-nm-diameter silica particles
PBS: phosphate-buffered saline
pI: isoelectric point
SDS-PAGE: sodium dodecyl sulfate- polyacrylamide gel electrophoresis.
